# Consequences of Online Misinformation on COVID-19: Two Potential Pathways and Disparity by eHealth Literacy

**DOI:** 10.3389/fpsyg.2022.783909

**Published:** 2022-02-14

**Authors:** Hye Kyung Kim, Edson C. Tandoc

**Affiliations:** Wee Kim Wee School of Communication and Information, College of Humanities, Arts, and Social Sciences, Nanyang Technological University, Singapore, Singapore

**Keywords:** COVID-19, ehealth literacy, online misinformation, preventive behavior, misinformed behavior

## Abstract

The COVID-19 pandemic poses an unprecedented threat to global human wellbeing, and the proliferation of online misinformation during this critical period amplifies the challenge. This study examines consequences of exposure to online misinformation about COVID-19 preventions. Using a three-wave panel survey involving 1,023 residents in Singapore, the study found that exposure to online misinformation prompts engagement in self-reported misinformed behaviors such as eating more garlic and regularly rinsing nose with saline, while discouraging evidence-based prevention behaviors such as social distancing. This study further identifies information overload and misperception on prevention as important mechanisms that link exposure to online misinformation and these outcomes. The effects of misinformation exposure differ by individuals’ eheath literacy level, suggesting the need for a health literacy education to minimize the counterproductive effects of misinformation online. This study contributes to theory-building in misinformation by addressing potential pathways of and disparity in its possible effects on behavior.

## Introduction

In a publicly televised press briefing sometime in April 2020, the former United States President Donald Trump suggested looking into injecting disinfectant as a potential way to treat the novel coronavirus disease 2019 (here after referred to as COVID-19). This was during the time when COVID-19 had already infected more than 800,000 and killed more than 40,000 in the United States. President Trump later clarified he was being sarcastic, but that did not stop some individuals from trying it. Right after the President’s remarks, New York City’s poison control center reported receiving calls related to exposure to bleach and disinfectant as doctors scrambled to warn people not to ingest disinfectants (Slotkin, 2020). Indeed, during the COVID-19 pandemic, medical experts around the world found themselves having to control the spread of not only the virus, but also misinformation about it. After declaring a global pandemic, the World Health Organization (WHO) also warned about an “infodemic” as misinformation on causes, remedies, and prevention spread online (Thomas, 2020). Fact-checking groups had to debunk viral posts, such as those claiming that eating garlic, consuming bananas, and gargling with saltwater can prevent or cure COVID-19.

Although studies and reports have documented ways that misinformation affected a range of behaviors, especially in the political context (Allcott and Gentzkow, 2017; Valenzuela et al., 2019), the effects of misinformation are particularly crucial to understand in the context of a pandemic, when individuals need reliable information to make critical decisions that can affect their own as well as others’ well-being. Governments around the world have asked their citizens to protect themselves and one another by engaging in preventive behaviors, such as handwashing and practicing safe distancing; and yet viral messages had also recommended preventive behaviors that medical experts had debunked as ineffective or even harmful. Exposed to a myriad of recommended behaviors—both reliable and unreliable—how did individuals behave in terms of prevention?

Using data from a three-wave panel survey conducted during the early stages of the COVID-19 pandemic, this current study documented adverse consequences of online misinformation exposure for people’s self-reported adoption of preventive behaviors. In doing so, this study advanced theoretical understanding of misinformation effects by addressing two potential mechanisms of how online misinformation exposure leads to negative outcomes, guided by the protection motivation theory (Rogers, 1975), and work on information overload (Schneider, 1987). To offer practical insights in communicating risk during a pandemic, this study also addressed ehealth literacy as an important boundary condition that determines an individual’s vulnerability to the negative impact of online misinformation.

Misinformation refers to the presence of objectively incorrect or false information, which is “not supported by clear evidence and expert opinion (Nyhan and Reifler, 2010).” Others use it as a term to encompass information that is completely fabricated as well as information that mixes truths and falsehoods to mislead others; this can be either “deliberately promoted or accidentally shared” (Southwell et al., 2018, p. 1). It is somewhat complicated to decide what is scientifically true or false, especially in a pandemic, as new scientific evidence emerges over time that may contradict previous beliefs and findings about the disease. In the current study, we classify as misinformation claims that have been debunked or corrected by expert scientific consensus as either completely false or misleading at the time of the study.

Misinformation can be either deliberately promoted or accidentally shared by individuals (Southwell et al., 2018) and the likelihood of propagation depends on a variety of factors that range from individual to community level factors, as well as to content specific factors (Southwell, 2013). Scholars have also scrutinized the role of social media platforms in the propagation of misinformation—not only do these platforms provide easy and accessible channels for misinformation to spread, but the logics behind social networking, such as prioritizing social relationship and mutual exchange, also provide an incentive to social media users to exchange humorous, outrageous, and popular content, sometimes at the expense of information quality (Duffy et al., 2019). The swarm of misinformation online, however, is not only due to humans sharing them—the misinformation ecosystem is also characterized by bots designed to spread falsehoods, hoaxes, and conspiracy theories (Wang et al., 2018; Jones, 2019).

While many studies have focused on political misinformation (Allcott and Gentzkow, 2017; Bakir and McStay, 2018; Farhall et al., 2019), another popular topic for misinformation is health, which can be seen in the volume of conspiracy theories and hoaxes about vaccination and cancer treatment, among others, being spread on social media (Oyeyemi et al., 2014; Dredze et al., 2016; Wang et al., 2019; Rodgers and Massac, 2020). A classic example of health-related misinformation is the false claim that the measles, mumps, rubella (MMR) vaccine causes autism. After the claim was initially published in 1998 (Wakefield et al., 1998), the scientific community refuted it (Taylor et al., 1999) and the author eventually retracted the publication. However, the false claim still influences many parents’ decision on child vaccination as evidenced by public health emergencies due to measles outbreak in the US and European countries in 2019 (The City of NY, 2019; World Health Organization, 2019).

In the context of COVID-19, an analysis of messages being forwarded on the messaging app WhatsApp in Singapore found that 35% of the forwarded messages were based on falsehoods, while another 20% mixed true and false information (Tandoc and Mak, 2020). Among different types of false claims on COVID-19, information concerning the prevention and treatment of COVID-19 poses a great threat to public health by misleading people to engage in ineffective and potentially harmful remedies. To counter the negative impact of misinformation, the WHO and government agencies across countries have monitored and clarified false or still not scientifically tested claims about COVID-19 prevention and cure.

Studies have examined the effects of misinformation on perceptions and information behaviors (e.g., sharing) (e.g., Guess et al., 2019; Valenzuela et al., 2019), as well as on self-reported preventive behaviors (Lee et al., 2020; Roozenbeek et al., 2020; Loomba et al., 2021). In the context of COVID-19, a cross-sectional survey of 1,049 South Koreans found an association between receiving misinformation and misinformation belief as well as between misinformation belief and the number of preventive behaviors performed (Lee et al., 2020). Using five national samples, Roozenbeek et al. (2020) also found a negative association between belief in COVID-19 misinformation and self-reported compliance with health guideline. A randomized controlled trial also found that exposure to online misinformation on COVID-19 vaccine reduced vaccination intention in the United Kingdom and United States (Loomba et al., 2021). Studies point to possible effects of online misinformation exposure; however, the indirect effects of online misinformation were not tested, when doing so can help to identify psychological mechanisms of misinformation effect. More importantly, there is a dearth of studies that address engagement in the behaviors advocated by misinformation (i.e., misinformed behaviors, such as eating garlic) beyond compliance to health guidelines (e.g., social distancing). One possibility is that the mechanism of how misinformation shapes misinformed behaviors differs from how misinformation affects evidence-based practices.

To fill these important gaps, we examined whether exposure to online misinformation on COVID-19 prevention influences self-reported engagement in misinformed behaviors as well as evidence-based practices advocated by health authorities. Individuals may not be as motivated to engage in evidence-based practices when they learn about alternative, misinformed remedies to protect themselves against COVID-19. A recent cross-sectional survey found that belief in the prevalence of misinformation on COVID-19 motivates people to comply with official guidelines (Hameleers et al., 2020). However, given that some individuals often do not evaluate the veracity of (mis) information online (Pennycook and Rand, 2019), misinformation exposure may negatively influence their evidence-based practices without them realizing that what they had considered as “evidence” was unreliable information (Loomba et al., 2021).

Understanding psychological mechanisms underlying the effect of online misinformation exposure on self-reported behaviors is important for theory development as well as for mitigating the negative effects of online misinformation. Informed by theories of behavior change and cognitive load, respectively, we explored two potential pathways: misperception on prevention and information overload.

Misperception refers to incorrect or false beliefs, which could be formed because of exposure to misinformation (Southwell et al., 2018). In the current context, misperception refers to incorrect response efficacy associated with a misinformed behavior as to whether adopting the behavior will be effective at reducing the risk of COVID-19. Researchers have addressed the challenge in correcting misinformation once misperceptions are formed (Lewandowsky et al., 2012; Pluviano et al., 2017; Walter and Tukachinsky, 2020). Individuals find messages consistent with their prior beliefs as more persuasive than counter-attitudinal messages, sometimes at the expense of accuracy (Lazer et al., 2018).

According to McGuire (1989), to change a belief or attitude, individuals need to be first exposed to relevant information, pay attention, and process the information. When the information is accepted and stored in memory, individuals retrieve that information when they make behavioral decisions. Research has shown that people often do not think critically about the veracity of information they encounter online (Pennycook and Rand, 2019). During a pandemic marked by uncertainty, individuals may be motivated to believe whatever possible measures they can take to protect themselves regardless of the veracity of the information they find.

The Protection Motivation Theory (Rogers, 1975) theorizes that believing in the effectiveness of a protective action in reducing the disease’s threat promotes actual engagement in the protective action. If individuals believe that misinformed behaviors such as eating garlic and regularly gargling with mouthwash can help reduce their risk of COVID-19, they will be more likely to engage in those behaviors. Indeed, one study found that believing in cancer misinformation (e.g., “indoor tanning is more dangerous than tanning outdoors”) reduced intention to perform the behavior (e.g., indoor tanning) (Tan et al., 2015). In this study, we examined whether perceived efficacy of prevention measures against COVID-19, regardless of whether they are scientifically proven or not, increases subsequent behavior.

Furthermore, early research on cognitive load has also shown that individuals have limitations in the amount of information they can process (Miller, 1960, 1994). Information overload occurs when the processing requirements exceed this processing capacity (Schneider, 1987). Information overload is defined as the state when an individual cannot properly process and utilize all the given information (Jones et al., 2004). It often involves the subjective feelings of stress, confusion, pressure, anxiety or low motivation (O’Reilly, 1980).

The quality of decisions correlates positively with the amount of information one receives but only up to a certain point (Chewning and Harrell, 1990). Beyond this critical point, additional information will no longer be integrated into making decisions and information overload will take place (O’Reilly, 1980), resulting in confusion and difficulty in recalling prior information (Schick et al., 1990). Besides the amount of information, the level of ambiguity and uncertainty also contribute to the likelihood of information overload (Keller and Staelin, 1987; Schneider, 1987). That is because individuals can more easily process high-quality information than low-quality or unclear information (Schneider, 1987). Given that online misinformation contains uncertain and novel information regarding the prevention of COVID-19, exposure to such information can increase information overload. Relatedly, a study found that exposure to misinformation led to information avoidance and less systematic processing of COVID-19 information (Kim et al., 2020).

With information overload, objective facts become less influential in shaping people’s perceptions and behaviors as people find it difficult to identify and select important information (Eppler and Mengis, 2004; Vogel, 2017). In health contexts, a study found that higher levels of information overload predicted higher levels of self-reported stress and poorer health status at a 6-weeks follow-up (Misra and Stokols, 2011). In another study, cancer information overload significantly decreased cancer screening behavior at 18-month follow-up (Jensen et al., 2014). As such, information overload may prevent individuals from finding and processing information regarding evidence-based practices on COVID-19 prevention.

Scholars have sought to explain why some individuals believe or act on inaccurate information online (Tandoc, 2019). A study suggested that the effect of COVID-19 misinformation varies across sociodemographic groups, based on gender, ethnicity, or employment status (Loomba et al., 2021). In another study, those who are less capable of processing basic numerical concepts (i.e., low numeracy) were found to be more susceptible to misinformation on COVID-19 (Roozenbeek et al., 2020). Researchers suggested that technological advancement has exacerbated health disparity, especially among more vulnerable groups (Parker et al., 2003), partly due to limited navigation skills required for those technologies (Cline and Haynes, 2001). Southwell (2013) also pointed out that individuals with more exposure to health misinformation have fewer chances or capabilities to correct such incorrect information. Building upon this line of work, we propose ehealth literacy as an important boundary condition for the effect of online misinformation on subsequent health behavioral decisions.

eHealth literacy refers to the capability to obtain, process, and understand health information from electronic sources to make appropriate health decisions (Swire-Thompson and Lazer, 2020). eHealth literacy is viewed as a process-oriented skill that advances overtime with the introduction of new technologies and changes in personal, social, and environmental contexts (Norman and Skinner, 2006b). Studies have shown that low health literacy leads to delay or not getting health care, poorer overall health status and knowledge, less health promoting behaviors, and higher mortality rates (Institute of Medicine, 2004; Berkman et al., 2011). Compared with high ehealth literates, those with low ehealth literacy level are less capable of discerning or analyzing the quality of online health information and its source (Norman and Skinner, 2006b), making them more likely to believe in misinformation. Prior studies also emphasized the role of cognitive ability—those who have low levels of analytical thinking or numeracy were more vulnerable to being misinformed (De keersmaecker and Roets, 2017; Pennycook and Rand, 2019; Roozenbeek et al., 2020). Also, with a smaller capacity to process information, low ehealth literates may suffer more from information overload upon exposure to misinformation online. Indeed, researchers have emphasized the importance of improving health information literacy to cope with information overload (Kim et al., 2007). More importantly, low ehealth literates may find it more difficult to verify misinformation from other information sources; they may also have lower capability to obtain the needed health information online. These would make them more likely to engage in misinformed behaviors.

**FIGURE 1:**
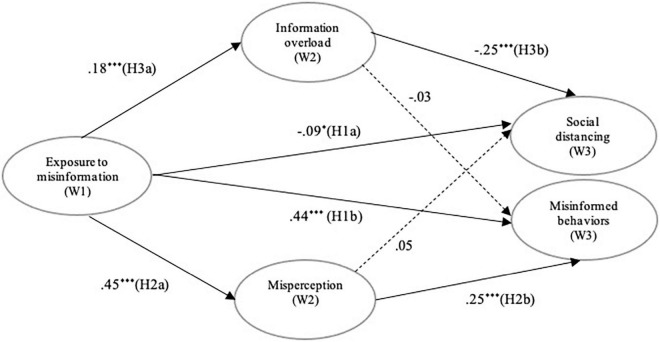
A structural model of online misinformation effects. Displayed values are standardized coefficients. Adjusted for age, gender, education, income, ethnicity. *Denotes *p*< 0.05, ***p< 0.001.

We aimed to investigate the effects of online misinformation exposure on two outcomes: self-reported adoption of evidence-based practices as well as misinformed behaviors during the COVID-19 pandemic. Specifically, we predicted that exposure to online misinformation on COVID-19 prevention will (H1a) decrease evidence-based prevention practices and (H1b) increase misinformed behaviors. Guided by the protection motivation theory (Rogers, 1975), and work on information overload (Schneider, 1987), we proposed two specific mechanisms for this impact. First, we predicted that (H2a) exposure to online misinformation will increase misperception on prevention; and that (H2b) misperception will increase engagement in misinformed behaviors. Second, we hypothesized that (H3a) exposure to online misinformation will increase information overload and that (H3b) information overload will reduce engagement in evidence-based prevention practices. Finally, this study posed a research question to examine the moderating role of ehealth literacy between the paths proposed in H1-H3: “Does the effect of online misinformation exposure differ based on levels of ehealth literacy?” (RQ1) Figure 1 summarizes our hypotheses.

## Materials and Methods

### Participants and Procedures

A three-wave online panel survey was conducted with Singaporeans recruited from an online panel managed by Qualtrics. Singapore, an urban, developed country in Southeast Asia, is one of the countries hit hard by COVID-19 in the early stages of the pandemic. The Wave 1 survey (*N* = 1,023 out of 1,182 who initiated) was conducted in February 2020 while the Wave 2 survey was conducted in March 2020 (*N* = 767 out of 827 who started; retention rate of 75.0%). In April 2020, 540 participants completed the Wave 3 survey (retention rate of 70.4% from Wave 2). The survey was administered in English and took about 15 minutes to complete. Each wave collected data for 2 weeks, and Qualtrics sequentially sent out email invitations to keep the time interval consistent across participants. Participants were incentivized based on Qualtrics’ remuneration system. During the data collection period, the local cases of COVID-19 increased from 96 to 8,014 in Singapore, recording among the highest number of cases in Asia at that time. We employed quota sampling based on age, gender, and ethnicity to achieve a sample that resembles the national profile. This study was approved by the Institutional Review Board of the hosting university in Singapore.

### Measures

To ensure the temporal ordering among study variables, we assessed exposure to misinformation at Wave 1, information overload and misperception at Wave 2 and engagement in social distancing and misinformed behaviors at Wave 3.

#### Exposure to Online Misinformation

We identified four false claims on how to prevent COVID-19: (1) gargling with mouthwash, (2) eating garlic, (3) vaccination against pneumonia, and (4) regularly rinsing nose with saline. These claims spread online during the early stages of the pandemic and were clarified as false by World Health Organization (2020). Based on prior research on exposure to scanned health (mis) information (Niederdeppe et al., 2007; Tan et al., 2015), the participants were asked how often they had heard in the past few weeks that each of the four behaviors can prevent COVID-19. For each false claim, we measured exposure from the following three online sources, separately (1 = not at all; 2 = a few times; 3 = several times: 4 = a lot of times): news app or website, social media app or website, medical or health websites. Following the approach taken in an earlier study on misinformation exposure (Tan et al., 2015), responses were averaged to create a scale of exposure to each of the four pieces of misinformation (gargling, α = 0.93, *M* = 1.33, *SD* = 0.68; garlic, α = 0.87, *M* = 1.33, *SD* = 0.62; vaccination, α = 0.93, *M* = 1.30, *SD* = 0.62; saline, α = 0.93, *M* = 1.20, *SD* = 0.56).

#### Information Overload

To assess the feelings of being overwhelmed with information on COVID-19, we used eight items derived from Jensen et al. (2014). Participants reported their agreement with each statement, such as, “There is not enough time to do all of the things recommended to prevent the COVID-19,” and “No one could actually do all of the COVID-19 recommendations that are given” (1 = strongly disagree; 5 = strongly agree; α = 0.87, *M* = 2.73, *SD* = 0.74).

#### Misperception

Misperception focused on the perceived efficacy of four misinformed behaviors. We thus asked to what extent participants thought each of the four misinformed behaviors were effective ways to reduce their risk of COVID-19 on a 5-point scale (1 = not at all effective; 5 = extremely effective). Misperceptions were measured with a single item for each misinformed preventive behavior (gargling, *M* = 2.03, *SD* = 1.11; garlic, *M* = 1.90, *SD* = 1.03; vaccination, *M* = 2.34, *SD* = 1.16; saline, *M* = 1.89, *SD* = 1.02). Responses to four items were reliable (α = 0.86, *M* = 2.04, *SD* = 0.91).

#### Social Distancing

For evidence-based practices, we focused on social distancing as this particular behavior was most actively promoted between Wave 2 and Wave 3 with the implementation of lockdown measures in Singapore (Yong, 2020). Participants self-reported how often they engaged in social distancing in the past 2 weeks on a 5-point scale (1 = never; 5 = very often). We used two items based on the advisories released by Ministry of Health in Singapore (gov.sg, 2020): (1) staying home as much as you can, and (2) keeping a safe distance from others (Spearman-Brown coefficient = 0.71, *M* = 4.67, *SD* = 0.54).

#### Misinformed Behaviors

For each of the misinformation items, we assessed the corresponding behavior: (1) gargling with mouthwash, (2) eating garlic, (3) vaccination against pneumonia, and (4) regularly rinsing nose with saline. Participants self-reported whether they had engaged in each of the behaviors in the past 2 weeks to prevent COVID-19 (0 = no, 1 = maybe, 2 = yes; α = 0.81, *M* = 0.28, *SD* = 0.48). When participants answered “yes,” they were asked to report the total number of times they did the respective behavior (except vaccination): gargling with mouthwash (*M* = 8.93, *SD* = 9.53, *range* = 1–60), eating garlic (*M* = 5.94, *SD* = 5.77, *range* = 1–24), and rinsing nose with saline (*M* = 4.26, *SD* = 6.38, *range* = 1–28).

#### eHealth Literacy

We used an 8-item ehealth literacy scale from Norman and Skinner (2006a). Participants reported their level of agreement with eight statements about their experience using the Internet for health information (1 = strongly disagree; 5 = strongly agree). Sample statements include: “I know what health resources are available on the Internet,” and “I know how to use the health information I find on the internet to help me” (α = 0.94, *M* = 3.80, *SD* = 0.68).

### Analytic Approach

We used structural equation modeling (SEM; AMOS 25) to estimate path coefficients while accounting for measurement errors by using latent variables (Aiken et al., 1994). We followed a two-step process of latent path modeling, which examines a measurement model first and then a structural path model. SEM is also recommended for group comparisons with latent variables as it allows statistical testing for group differences in path coefficients (Cole et al., 1993; Aiken et al., 1994). For group comparisons, we used the median split to categorize participants into either the high or low ehealth literacy group. All constructs were treated as latent variables with respective measurements. We used item parceling with a random algorithm, for a latent factor with more than six indicators, because parceling helps to remove theoretically unimportant noise (Matsunaga, 2008). We employed the full information maximum likelihood (FIML) method to address missing data (Graham, 2009). We controlled for age, gender, education, and income in model testing.

## Results

### Sample Profile

Participants were restricted to those aged 21 or older and they were on average 44 years old (*SD* = 12.43) and 51.7% male at Wave 1 (Table 1). The majority of respondents were ethnic Chinese (80.4%), followed by 10.5% Malay, 6.4% Indian, 0.8% Eurasian and other race (2.1%). The median education attainment was university graduate and median household monthly income was in the range of SGD 6,000–7,999 (equivalent to USD 4,283–5,711). The attrition rate differed between genders from Wave 2 to Wave 3, *p* = 0.03 and among those who received different levels of formal education from Wave 1 to Wave 2, *p* = 0.01. The robustness check using the balanced samples of who completed all waves found the same results as those using the imputed data employing FIML estimation.

**TABLE 1:** Sample profile.

	Wave 1 (*N* = 1,023)	Wave 2 (*N* = 767)	Wave 3 (*N* = 540)
	*M* (*SD*) or%	*M* (*SD*) or%	*M* (*SD*) or%
Age	43.79 (12.43)	44.26 (12.31)	44.91 (12.26)
Gender (Male)	51.7%	52.0%	54.6%
Ethnicity (Chinese)	80.4%	84.5%	86.3%
Malay	10.5%	8.3%	8.0%
Indian	6.4%	4.8%	4.1%
Eurasian	0.8%	0.7%	0.6%
Other	2.1%	1.7%	1.1%
Education (upper secondary or less)	15.2%	12.3%	12.4%
Junior college, pre-university, polytech	29.1%	30.2%	29.4%
University	44.8%	45.9%	47.4%
Graduate/professional degree	10.9%	11.6%	10.7%
Monthly income (SGD) (below 3,999)	23.6%	19.6%	17.6%
4,000–7,999	32.6%	35.5%	35.9%
8,000–11,999	24.5%	24.6%	25.6%
12,000 and above	19.3%	20.4%	20.9%
			

### Confirmatory Factor Analysis

Using confirmatory factory analysis (CFA), we first validated the measurement model with all latent factors in the proposed model. A good model has a root mean square error of approximation (RMSEA) ≤ 0.06, a comparative fit index (CFI) ≥ 0.95, and a standardized root mean square residual (SRMR) < 0.08 (Hu and Bentler, 1999). The CFA model fitted satisfactorily (χ^2^/df = 3.28, CFI = 0.97, RMSEA = 0.047, SRMR = 0.029). As presented in Table 2, standardized loadings for all factors ranged from 0.60 to 0.93 (Kline, 2011). The composite reliabilities (CRs) of latent variables were all above 0.7 and the average variance extracted (AVE) values of the latent factors were all above 0.5 (Hair et al., 2010). The square root of each construct’s AVE was also greater than its correlation with other latent factors. Thus, the CFA model had sufficient reliability, as well as convergent and discriminant validity (see Table 3).

**TABLE 2:** Descriptive statistics and estimates of measurement model.

Constructs	Items	*M (SD)*	*Standard loadings*	α
Online misinformation exposure	Gargling with mouthwash	1.33 (0.68)	0.79	0.90
	Eating garlic	1.33 (0.62)	0.83	
	Vaccination against pneumonia	1.30 (0.62)	0.83	
	Regularly rinsing with saline	1.20 (0.56)	0.90	
Information overload	Parcel 1^a^	2.54 (0.71)	0.93	0.84
	Parcel 2^b^	2.93 (0.69)	0.78	
	Parcel 3^c^	2.74 (0.82)	0.72	
Misperception	Gargling with mouthwash prevents COVID-19	2.03 (1.11)	0.87	0.86
	Eating garlic prevents COVID-19	1.90 (1.03)	0.91	
	Vaccination against pneumonia prevents COVID-19	2.34 (1.16)	0.79	
	Regularly rinsing with saline prevents COVID-19	1.89 (1.02)	0.60	
Misinformed behaviors	Gargling with mouthwash	1.38 (0.53)	0.82	0.81
	Eating garlic	1.34 (0.49)	0.72	
	Vaccination against pneumonia	1.23 (0.41)	0.75	
	Regularly rinsing with saline	1.18 (0.38)	0.73	
Evidence based practice	Staying home as much as you can	4.67 (0.47)	0.65	0.71^d^
	Keeping a safe distance from others	4.67 (0.43)	0.86	

*^a^Parcel 1: (1) There are so many different recommendations about preventing the COVID-19, it’s hard to know which ones to follow, (2) It has gotten to the point where I don’t even care to hear new information about the COVID-19, (3) I forget most the COVID-19 information right after I hear it.*

*^b^Parcel 2: (1) Information about the COVID-19 all starts to sound the same after a while, (2) Most things I hear or read about the COVID-19 seem pretty far-fetched, (3) I feel overloaded by the amount of the COVID-19 information I am supposed to know.*

*^c^Parcel 3: (1) There is not enough time to do all of the things recommended to prevent the COVID-19, (2) No one could actually do all of the COVID-19 recommendations that are given.*

*^d^Spearman-Brown coefficient.*

**TABLE 3:** CR, AVE, and correlations among the latent constructs.

	CR	AVE	1	2	3	4	5
1. Misinformation (W1)	0.91	0.71	**0.84**				
2. Information overload (W2)	0.85	0.66	0.17	**0.81**			
3. Misperception (W2)	0.88	0.65	0.45	0.22	**0.80**		
4. Social distancing (W3)	0.73	0.58	−0.11	−0.26	−0.04	**0.76**	
5. Misinformed behaviors (W3)	0.84	0.57	0.54	0.10	0.44	−0.06	**0.76**

*Diagonal elements (bold text) are the square root of the AVE for each construct.*

### Structural Model

The baseline model with all groups adequately explained patterns of association between latent constructs (χ^2^/df = 2.85, CFI = 0.96, RMSEA = 0.042, SRMR = 0.039). As shown in Figure 1, exposure to online misinformation at Wave 1 significantly reduced engagement in social distancing [β = −0.09, *p* = 0.032, 95% CI (−0.18, −0.02)] and increased misinformed behaviors [β = 0.44, *p* < 0.001, 95% CI (0.35, 0.52)] at Wave 3 (H1 supported). Exposure to online misinformation at Wave 1 led to greater misperception [β = 0.45, 95% CI (0.38, 0.51); H2a supported] and information overload [β = 0.18, 95% CI (0.11, 0.24); H3a supported] at Wave 2 (both *p* < 0.001). Misperception at Wave 2 significantly increased subsequent misinformed behaviors [β = 0.25, *p* < 0.001, 95% CI (0.16, 0.34); H2b supported], while it did not change social distancing behavior at Wave 3 [β = 0.05, *p* = 0.45, 95% CI (−0.06, 0.12)]. As predicted, information overload significantly reduced social distancing [β = −0.25, *p* < 0.001, 95% CI (−0.32, −0.18); H3b supported], while having no effect on misinformed behaviors [β = −0.03, *p* = 0.50, 95% CI (−0.10, 0.04)].

We conducted bootstrapping for mediation analyses. Bootstrapped confidence intervals (at the.05 level with 5,000 re-samples) showed a significant indirect effect of exposure to misinformation on misinformed behaviors [95% CI (0.037, 0.084)], but not on social distancing [95% CI (−0.045, 0.016)].

### Structural Model Invariance Test

To compare the path coefficients in the model between high and low ehealth literacy groups (RQ1), we conducted a structural model invariance test with cross-validation. The model with metric invariance constraints (i.e., keeping the measurement loadings constant across groups) had a good fit: χ^2^/df = 2.20, CFI = 0.95, RMSEA = 0.03, SRMR = 0.046. Thus, the CFA model is valid across the two groups, allowing us to compare the strength of path coefficients using these measures between groups (Byrne, 2001). Next, we compared between the baseline model and the models with cross-group equality constraints (i.e., keeping constant path coefficients between groups) using chi-square change as a statistical test. This procedure tests if model fit improves with a model that allows a path coefficient to change between groups. Thus, significant model fit improvement would be interpreted as having different path coefficient strengths between the low and high ehealth literacy groups. The effect of online misinformation exposure at Wave 1 on social distancing at Wave 3 was stronger in the low ehealth literacy group (β = −0.20, *p* < 0.001) than the high ehealth literacy group (β = −0.12, *p* = 0.07), Δχ^2^ (1) = 6.85, *p* = 0.001. Also, misperception at Wave 2 had a stronger effect on the performance of misinformed behaviors at Wave 3 in the low ehealth literacy group (β = 0.34, *p* < 0.001) than in the high ehealth literacy group (β = 0.17, *p* = 0.001). However, this group difference did not reach the statistical significance, Δχ^2^ (1) = 3.34, *p* = 0.068. No other paths in the proposed model differed by ehealth literacy (Table 4).

**TABLE 4:** Structural model invariance test.

Path	High ehealth literacy	Low ehealth literacy	Δχ^2^ (1)
Misinformation exposure → information overload →	0.25***	0.15**	0.05
Misinformation exposure → misperception	0.54***	0.21***	2.58
Information overload → social distancing	−0.29***	−0.18***	0.11
Misperception → misinformed behaviors	0.17**	0.34***	3.34^†^
Misinformation exposure → social distancing	–0.12	−0.20***	6.85**
Misinformation exposure → misinformed behaviors	0.50***	0.34***	0.15
Information overload → misinformed behaviors	–0.03	0.02	0.66
Misperception → social distancing	0.11	0.003	1.11

Standardized coefficients **denotes p < 0.01, ***p < 0.001, ^†^p < 0.10.

## Discussion

This study found that exposure to online misinformation reduced self-reported engagement in social distancing and increased misinformed behaviors. This effect was partly explained by greater misperception and information overload triggered by online misinformation exposure. Misperception increased subsequent misinformed behaviors, while information overload reduced social distancing. Moreover, the effects of misinformation exposure differed by individuals’ eheath literacy level. We discuss theoretical and practical implications of these key findings.

Exposure to online misinformation on COVID-19 prevention not only increased self-reported engagement in behaviors advocated in the misinformation, but it also discouraged engagement in social distancing, which had been reinforced by health and government authorities during the pandemic. This suggests that misinformation exposure can discourage the adoption of evidence-based practices even when the content is not specifically on those practices. It is noteworthy that the two behavioral measures were not correlated (*p* = 0.70), indicating that they are independent from one another and that engaging in one behavior type may not compensate nor negate the other behavior. This echoes the differential mechanisms that led to the two different behavioral types found in this study.

Consistent with prior research (e.g., Nyhan and Reifler, 2010; Kishore et al., 2011; Wang et al., 2019), we found that exposure to misinformation can lead to misperception; in this specific context, individuals frequently exposed to online misinformation about preventive behaviors were more likely to believe in the efficacy of these misinformed behaviors at preventing COVID-19. Belief may be a default position when people encounter new information, and that disbelief requires mental effort, which people often do not bother to exercise when using online platforms (Pennycook and Rand, 2019). This is of concern given that some misinformation on COVID-19 promote fake remedies that can bring about detrimental health outcomes as seen in the cases of ingesting methanol and disinfectant to prevent COVID-19 (Associated Press, 2020; Slotkin, 2020).

We conceptualized misperception as perceived response efficacy of preventive measures based on PMT (Rogers, 1975), and, as the theory predicts, we found that misperception on COVID-19 prevention subsequently increased the likelihood of misinformed behaviors. Furthermore, a significant indirect effect of online misinformation exposure was found on prompting self-reported misinformed behaviors *via* cultivating misperception. Similarly, Tan et al. (2015) found that misperception on cancer causes, conceptualized as outcome expectancies, predicted intention to engage in cancer-related behaviors. Both response efficacy and outcome expectancies have been suggested as key motivators for health behaviors (Floyd et al., 2006; Fishbein and Ajzen, 2010). This highlights the importance of correcting misperception by addressing its scientifically ungrounded nature before individuals act on their incorrect beliefs. However, considering the difficulty in rectifying misperception (Lewandowsky et al., 2012; Pluviano et al., 2017), it would be also critical to minimize the public’s exposure to false claims on the internet. It may as well be important to build a critical mindset in dealing with online health information *via* inoculation or other prebunking strategies (van der Linden et al., 2020).

We also found that online misinformation exposure increased information overload. The uncertain and novel nature of misinformation on COVID-19 prevention might have been difficult to process, which made individuals feel more overloaded with information (Schneider, 1987). Information overload also reduced self-reported compliance to social distancing measures in line with prior research that found poorer health status and less engagement in cancer screening due to information overload (Misra and Stokols, 2011; Jensen et al., 2014). Objective information on preventive measures may become less influential when people experience information overload with the difficulty in identifying and selecting important information (Eppler and Mengis, 2004). Because failing to implement evidence-based practices can exacerbate the spread of a disease, we call for more research on the link between misinformation and information overload and other possible mechanisms to better explain the effects of online misinformation.

This study also explored the possible moderating role of ehealth literacy in how online misinformation exposure leads to self-reported preventive behaviors. We found one path in the proposed model that significantly differed between the low and high ehealth literacy groups. Specifically, the negative effects of online misinformation exposure on engaging in social distancing was stronger among those with lower levels of ehealth literacy than higher ehealth literates. Similarly, misperception also had a stronger, albeit marginal, effect on self-reported misinformed behaviors in the low ehealth literacy group. Collectively, it appears that online misinformation cultivates misperception regardless of ehealth literacy; however, high ehealth literates (vs. low literates) are less likely to act on their misperceptions perhaps because they are more capable of verifying and correcting their misperceptions over the course. This points to the value of ehealth literacy in empowering individuals in navigating an online information environment that has been polluted by different forms of health-related misinformation.

## Limitations

There are several limitations of this study worth noting. First, we focused on examining the impact of exposure to misinformation, but not exposure to correct information nor exposure to mixed messages while individuals often encounter information that includes both true and false claims (Brennen et al., 2020; Tandoc and Mak, 2020). Also, given that new scientific evidence and misinformation emerge as the pandemic develops, future work should examine other types of misinformation (e.g., on COVID-19 vaccines) to replicate our findings and to identify the most critical types of online misinformation during the pandemic.

Second, we relied on self-reported measures of exposure to online misinformation and behaviors, which means our data relied heavily on the capability and willingness of participants to correctly recall and self-report their exposure levels and behavior. Given the lack of empirical evidence on how misinformation influences the way people actually behave, future studies should employ observational designs to assess preventive as well as misinformed behaviors. While our exposure measures had good reliability and convergent/discriminant validity in the current study, future studies could develop and adopt alternative measures or manipulation of information exposure to avoid issues with self-report. Because we averaged misinformation exposure across three different online sources for the sake of model parsimony, future work could also consider treating this factor differently, for example, by modeling misinformation exposure separately by source type.

Third, we cannot confirm causal relations between study variables even with the temporal ordering established with the three-wave panel design. We collected data in the middle of a rapidly changing pandemic, which also involved constant updates on preventive measures advocated by health authorities. Such changes made it difficult for us to constantly assess study variables across three waves and control for factors measured in earlier waves, which would have provided more convincing causal evidence on misinformation effect. Also, our data only reflects the earlier stages of the outbreak in Singapore; nonetheless, we believe that it is crucial to examine misinformation effects during early stages of an actual pandemic, especially one that involves a novel disease, given that the spread of online misinformation is more frequent in such case as the public and scientific community struggle to figure out what it is and how to deal with the novel outbreak.

## Conclusion

Using a three-wave panel survey, this study offered some evidence that online misinformation exposure can lead to the public’s maladaptive behaviors during a disease pandemic. Furthermore, we addressed two types of behavioral responses to the pandemic with differential mechanisms through which exposure to online misinformation could prompt those behavioral responses. This study also provided initial evidence on the impact of online misinformation on information overload beyond misperception; thus, this study informs further theory development in online misinformation exposure and effects. Lastly, we identified ehealth literacy as a potential boundary condition for the adverse consequences of online misinformation exposure, which highlights the importance of health literacy education to fight the growing problem of misinformation online.

## Data Availability Statement

The raw data supporting the conclusions of this article will be made available by the authors, without undue reservation.

## Ethics Statement

The studies involving human participants were reviewed and approved by the Institutional Review Board of Nanyang Technological University. The participants provided their written informed consent to participate in this study.

## Author Contributions

HK and ET contributed to conception and design of the study. HK performed the statistical analysis and wrote the first draft of the manuscript. ET wrote sections of the manuscript. Both authors contributed to manuscript revision, read, and approved the submitted version.

## Conflict of Interest

The authors declare that the research was conducted in the absence of any commercial or financial relationships that could be construed as a potential conflict of interest.

## Publisher’s Note

All claims expressed in this article are solely those of the authors and do not necessarily represent those of their affiliated organizations, or those of the publisher, the editors and the reviewers. Any product that may be evaluated in this article, or claim that may be made by its manufacturer, is not guaranteed or endorsed by the publisher.
